# Biography Writing in the Pediatric Palliative Care Context: Review and Feasibility Data

**DOI:** 10.3390/children12010004

**Published:** 2024-12-24

**Authors:** Sarah Potter, Sandra Coombs, Tiina Jaaniste

**Affiliations:** 1 Department of Palliative Care, Sydney Children’s Hospital, Randwick, NSW 2031, Australia; sandra.coombs@health.nsw.gov.au (S.C.); tiina.jaaniste@health.nsw.gov.au (T.J.); 2 Department of Palliative Care, Children’s Hospital at Westmead, Westmead NSW 2145, Australia; 3 School of Clinical Medicine (Paediatrics and Child Health), University of New South Wales, Sydney, NSW 2052, Australia

**Keywords:** biography writing, pediatric, palliative care, meaning, volunteers

## Abstract

Background: Biography writing services are increasingly being used with adult palliative care patients, helping them document their stories in a way that provides meaning for themselves, family, and friends. However, the feasibility of applying a biography program to a pediatric palliative care context is less well known. Methods: A narrative review of the literature was conducted, drawing on existing adult biography programs, while noting differences between the pediatric and adult contexts. The Story Project is outlined in this manuscript as an example of a pediatric biography writing approach, with pilot survey data from six volunteer biographers presented. Thematic analysis was conducted on qualitative data elicited from volunteer biographers regarding challenges experienced and the perceived benefits of the program. Descriptive information is provided for other aspects of their experience of the Story Project. Results: The challenges identified by the volunteer biographers were grouped into seven themes, namely, (1) delays encountered, (2) rapport/interactions with family, (3) family distress, (4) logistics, (5) transcribing-specific issues, (6) identifying themes, and (7) finalizing the biography. The perceived benefits of the Story Project, as perceived by the volunteer biographers, were grouped into four main themes: (1) beneficial processes for families, (2) benefits for the volunteers, (3) intrinsic value of the final product, and (4) beneficial uses of the product. Biographies took between 3–20 months to complete, with most taking 12 months or less. Conclusions: Biography writing is a potentially valuable approach for use with pediatric palliative care patients and their families, with the Story Project being one example of such an intervention, with promising early feasibility data.

## 1. Introduction

Pediatric palliative care is a holistic, family-centered approach that aims to alleviate the physical, emotional, spiritual, or social suffering of patients and their families [1]. Existential distress often arises when individuals confront their own impending death or the death of a loved one [2]. To date, there are relatively few palliative care interventions or approaches that address existential distress [3,4]. Within the adult palliative care context, biography writing approaches have increasingly been used with patients to help them reflect and consider their collection of life stories [5,6,7]. It has been argued that such approaches help individuals to create meaning through the act of reflection and storytelling [3,8,9,10]. Less attention has been given to the use of biographical approaches in the pediatric palliative care context. Caring for a child and their family at the end of life requires approaches with the potential to alleviate or minimize existential distress and to find meaning in difficult circumstances.

This manuscript reviews the available literature pertaining to the use of biographical approaches in the context of palliative care. Approaches that have been used within the adult palliative care context are reviewed before a discussion of how the key elements of biographical approaches hold potential merit for the pediatric palliative care context. A small number of biographical interventions that have been used in the pediatric palliative care context are described. This is followed by a presentation of a pilot study examining the feasibility of one such pediatric biography writing approach, namely, the Story Project. Pilot data from volunteer biographers involved in the Story Project are presented, shedding light on the challenges and perceived benefits of this approach, as identified by volunteer biographers working in the pediatric palliative care context.

### 1.1. Biographical Approaches in the Palliative Care Context

Biographical approaches focus on exploring some form of life story. A comprehensive and scholarly overview of biographic methods across diverse fields such as sociology, psychology, history, literature, and anthropology has been provided by Bornat (2008) [11]. She argued that biographical approaches, in whatever form they take, promote understanding and interpretation of experience. It has been noted that “Narratives do not mirror, they refract the past… storytellers interpret the past rather than reproduce it as it was.” [12]. Bornat (2008) identified three key themes involved in biographical approaches, namely, (i) the interactive nature by which stories are elicited, (ii) the subjective expression of self, revealed through the sharing of one’s perceptions and understanding of circumstances and experiences, and (iii) the structure by which the stories and ideas are assembled, with a beginning, middle, and end [11].

Illness narratives have been used as a therapeutic tool for many decades [13,14,15], allowing patients to bring focus to their subjective experience of the illness rather than just the clinical experience [13]. A serious illness can change the biographical trajectory of the patient and family, resulting in the patient (if not the whole family) needing to renegotiate their sense of self identity [16]. Illness narratives have been used to help patients narrate their subjective illness experience to combat the depersonalization that may accompany a serious illness [17]. Illness narratives express an individual’s personal experience in the patient’s own voice, in contrast to a medical history, which typically incorporates a collection of facts based on a clinical account.

Biographical approaches began to gain traction in the context of end-of-life care from the 1970s, largely due to the effort of pioneers such as Cicely Saunders, who emphasized the importance of understanding life stories and personal histories in providing holistic care [18]. There has also been growing recognition that the process of storytelling holds intrinsic value for the individual, enabling them to reflect and explore personal issues, potentially acknowledging and addressing sources of tension [19,20]. Recounting one’s story or collection of stories can help individuals find personal meaning [3,10,19], preserve their sense of integrity [19,21], and potentially help them to better adjust to the reality of their situation [19].

The act of creating a tangible biography aligns with the notion of generativity, whereby individuals invest in “forms of life and work that will outlive the self” [22] (p. 10). The concept of generativity is particularly relevant in palliative care, as individuals reflect on the world they are leaving behind [21]. A recent study indicates that generativity plays a significant role in accepting death by enhancing ego-integrity [21]. Ego-integrity can be defined as recognizing and accepting oneself and one’s life journey, including both triumphs and disappointments [21]. When individuals feel they have successfully passed on what is meaningful to them, they experience fewer regrets, increased ego-integrity, and a greater acceptance of their approaching death [21].

Various biographical approaches have been used in the adult palliative care context [3]. The most widely used and validated approach is known as dignity therapy, with a 2021 systematic review citing 28 studies using dignity therapy in the palliative care context [5]. This therapy is designed to preserve the dignity of individuals who are dying by addressing the sources of their psychosocial and existential distress [23,24]. The dignity therapy approach outlined by Chochinov consists of a 30–60 min session in which a therapist asks a series of open-ended questions designed to encourage the patient to reflect and talk about their lives and what matters most to them [23,24,25]. The conversation is recorded, transcribed, and edited within a few days. The patient is then provided an opportunity to review the document and make any changes before the final document is produced. The patient may then share the document with family and friends as they wish.

An unstructured biographical intervention in the adult palliative care context, conducted by trained volunteers, has also been described in the literature [6]. Patients were given the option of sharing their stories in one or two interview sessions; however, all opted to tell their story in a single session, with interviews lasting between 1 and 2 h. These were then transcribed, and the stories were handed back to the patient on average 11 days after the interview [6].

### 1.2. Biography Writing in the Pediatric Palliative Care Context

While there has been significantly less research on biographical approaches in the pediatric palliative care context relative to the adult palliative care context, the approach has considerable merit. Pediatric and young adult illness narratives have been published in the context of various serious illnesses [26,27]. Potential benefits arise from how the patient and/or their family use the final biography document. However, benefits may also stem from the process of self-reflection and sharing that goes into preparing the biography document. Each of these aspects are examined in this paper in more detail, specifically within pediatric palliative care context.

#### 1.2.1. Utilization of the Final Biography Book

Biography books created to reflect on the life of a child or adolescent who is expected to die relatively soon, or who has already died, can be used by family members in various meaningful ways. Caregivers or family members may use the book as a tangible resource to help them reflect on the life of their child. Additionally, parents commonly wish for their child’s life to be remembered by others, and a biography book can provide a way to share their child’s story and life journey. Notably, “stories can provide a window into their experience…” [19] (p. 16). This may be particularly valuable if a child was non-verbal and it was difficult for others outside of the family to get to know the child’s personality.

Biography books may be created for current or future use by siblings. Some siblings may not fully understand the situation or the impending death of their brother or sister, especially if they are very young. Biography books may be created in simple age-appropriate language to be something like a story book about the life of their sick brother or sister and their sibling relationships as well as relationships with other family members. These biography books may be used to promote sibling understanding and to provide opportunities for discussion with others. Anecdotally, parents sometimes worry that young siblings will not remember their sick brother or sister after they have died. Biography books may help siblings as they get older to better understand events and to consolidate their early memories of their brother or sister.

Biography books are sometimes primarily created for the young patient. These may be something that the young person wishes to gift or leave behind as a legacy for their family and friends. A study by Akard et al. (2013) sought to determine what children with advanced cancer wished for other to know or remember about them [28]. Their qualitative analysis revealed three broad areas which these children wanted others to know or remember. First, they wanted their personal and unique characteristics to be remembered. Second, they wanted others to remember the things they liked to do. Third, they wanted their connectedness with others to be remembered. Development of a biography document can potentially help young people achieve these goals.

#### 1.2.2. The Process of Creating a Biography

Although family members may enter the biography program focused on achieving the end product (i.e., the biography book), the process of reflecting on what is significant in their child’s life in order to create the final document may itself be highly valuable. Documenting what has been significant and meaningful in the young person’s life may encourage a greater focus on their personhood rather than just on their illness and daily treatment requirements, which can occupy a big part of a caregiver’s focus.

Verbal patients, especially adolescents, may find the process of documenting their story and shaping how others will remember them to be valuable. Parents of children with advanced cancer participating in a digital storytelling project reported that the intervention helped their child to express their feelings in addition to providing the parents with emotional comfort [29]. Moreover, engaging in creative processes may serve as a distraction from their symptoms, with potential benefits for quality of life [30,31].

### 1.3. Pediatric Biographical Interventions in the Palliative Care Context

Very little research exists regarding the use of biographical approaches in the pediatric end-of-life context. Nevertheless, legacy-making or memory-making interventions are commonly used by clinicians working in the field of pediatric palliative care [32,33], which may involve plaster molds of a child’s hand or foot, video recordings, online writing, or written documents. Akard et al. (2015) developed a digital storytelling intervention for children [29]. Participants were given the opportunity to share how they wanted to be remembered or known using audio, video, music, and photographs, with the information compiled into a final digital product [29].

Steps have been taken to adapt the adult dignity therapy approach to adolescent palliative care patients [34,35]. A series of 12 interview questions were developed for use with adolescents, with these being designed to align with the core concepts of the dignity therapy approach [34]. These questions formed the basis of a 30–60 min interview and were trialed with a group of healthy Portuguese adolescents. The adolescents deemed the questions clear and unambiguous, expressing that the interview would have particular relevance for adolescents with life-limiting conditions [34].

A second group of North American researchers assessed the feasibility of creating dignity therapy biography-based documents in a pediatric palliative care context [35]. They trialed the intervention with eight patients ranging in age from 2 months through to 22 years. In four cases, the documents were created by the patients themselves. For those who were pre-verbal or non-verbal, caregivers helped created the dignity therapy documents. The first half of the interview focused on major life events and special memories, whereas the second half explored “any wisdom, wishes, or important words that still need to be said” [35] (p. 157). The authors inferred that the intervention was feasible since all the initiated interventions were completed. Adolescent and young adult participants expressed a desire not to be confined to the traditional text-only document, as commonly generated with adult patients [36]. Instead, they felt it to be important to include elements such as photographs and original artwork [35].

### 1.4. Personnel for Biography Writing Approaches

Within the palliative care literature, biography writing approaches have been facilitated by varying personnel. Dignity therapy interventions are typically conducted by health professionals specializing in palliative care [5]. The manualized intervention described by Chochinov et al. (2005) was implemented by a psychiatrist or psychologist and palliative care nurses [23]. A series of case studies illustrating the use of dignity therapy with pediatric patients and/or their families have described interventions delivered by a physician and a grief and bereavement specialist [35]. A pediatric digital storytelling intervention used trained nurses and a videographer [29]. Other adult biography writing projects have used trained volunteers [6,7] or students from a community-based narrative storytelling course [37]. Another biography intervention delivered to elderly people living in a residential aged-care setting used students trained in interviewing, the biography writing process, and Karuna Buddhist principles [38].

When setting up new biography writing programs, a number of factors need be considered when making decisions about personnel. A careful analysis of the biographer role and necessary skill set is needed to determine whether it is possible to adequately train volunteers for the role or whether more specific healthcare qualifications are required. Furthermore, an understanding of the time commitments and budgetary constraints is needed. Consistent with the studies described above, the use of more highly qualified personnel is achievable for programs that are of shorter duration, such as dignity therapy interventions. In contrast, volunteers may be able to devote more extended amounts of time, thereby facilitating lengthier interventions.

### 1.5. Aims

Given the potential value of biography writing approaches in the pediatric palliative care context, there is a need for specific documentation about potential approaches, with information about the feasibility of such approaches. The current study had four main aims. The first aim was to document the perceptions of volunteer biographers about the training they received to prepare them for their role in a biography writing program (namely, the Story Project) within the pediatric palliative care context. Secondly, we aimed to identify the challenges experienced by volunteer biographers in their role. Thirdly, we aimed to identify the perceived benefits of the Story Project, as reported by the volunteer biographers. The fourth aim was to document the specific details of each volunteer’s most recent biographies (such as time taken, number of sessions with family members, the people who contributed to the biographies, any delays encountered, and the number of unique biography books completed for the family). It is beyond the scope of the current manuscript to explore the specific content of biography documents produced through the Story Project.

## 2. Materials and Methods

### 2.1. Design

A cross-sectional, survey-based study was conducted. This study was ethically approved by the SCHN Quality Improvement Ethics Committee (approval number: QIE-2024-12-24).

### 2.2. Participants

Participants were volunteers currently working as biographers in the Pediatric Biography Program (Story Project) in the Sydney Children’s Hospital Network (SCHN) Palliative Care Services. There were no exclusion criteria. All six volunteer biographers who were currently engaged with the program agreed to participate in this study.

### 2.3. Procedure

Informed consent was requested from potential participants. Participants were then emailed a survey link (via the Teams platform) with questions about their experiences as a volunteer biographer in the SCHN pediatric palliative care services. Data collection occurred in September–October 2024.

#### Intervention: The Story Project

The Story Project is a biography writing intervention designed for use in the pediatric palliative care context with patients and/or their families. Work began on developing the Story Project at Sydney Children’s Hospitals Network in the second half of 2019. A steering committee was established comprising volunteer managers, palliative care clinicians (medical, nursing, and allied health), and a quality manager to establish structure and processes. Recruitment and training of volunteer biographers occurred in 2021, with family interviews commencing in mid-2021. The first completed biography was completed and presented to the family at the end of 2021.

The key steps involved in the implementation of the Story Project within the context of a pediatric palliative care service are outlined in Table 1.

To date, there have been 21 families who have been involved in the SCHN Story Project, resulting in completed biographies for 13 families, with other biographies either in progress or stalled. There have been 7 volunteer biographers involved with the Story Project throughout the 4-year history of the program. In light of the very limited published information about the use of biographical approaches in the pediatric palliative care context, it would be valuable to document the experiences and challenges of individuals involved in facilitating this program.

### 2.4. Measures

The survey was developed for the purposes of the current study and ongoing quality improvement. The survey included quantitative and qualitative questions, asking participants for feedback about their experiences and perceptions of volunteering in the Story Project. Information was elicited on the following topics: adequacy of training received, challenges in the interview phase and in the transcribing/editing phase, perceived positive outcomes associated with the program, specific details regarding their last completed biography project, occurrence and reasons for any delays, and satisfaction with the process and with the final product.

### 2.5. Analyses

Basic descriptive analyses were carried out for the quantitative data obtained. Qualitative responses were coded using methods of thematic analysis [39]. Specifically, thematic analysis was used to analyze participant responses about the nature of (i) any challenges that were experienced in their role as volunteer biographers and (ii) any perceived positive outcomes. The following steps were taken: (1) Two coders familiarized themselves with the data (TJ and SC). (2) One coder (TJ) generated initial codes through analysis of the data. (3) Codes were grouped into themes by this coder. (4) These themes were then refined through an iterative process of discussion with the second coder (SC) to ensure they accurately represented the nuances of the data. (5) Illustrative quotations were selected from the participant responses to support each theme and illustrate the breadth of responses within each theme.

## 3. Results

### 3.1. Sample Descriptives

Of the six volunteer biographers, five were female and one was male. Volunteers with the program were retired individuals, ranging between 58 and 71 years in age. Several volunteers were previously from professional careers (e.g., lawyers, nurses), and some were previously freelance writers or former literacy teachers. At the time of survey completion, three volunteers were actively working with a family on a biography project, two were awaiting assignment to their next family, and one volunteer was having a planned break between biography projects.

### 3.2. Volunteer Perceptions About Training

All six respondents reported that they had received enough training for the role. When asked to rate the helpfulness of the training on a 0–10 scale, three volunteers reported 10, two reported 8, and one reported 7. It was suggested that volunteers could be advised up front that the process of creating a biography with a patient or family may be a lengthy process. Several volunteers noted that the lessons learnt since the start of the program should be incorporated into the training of new volunteers, potentially with talks from volunteers and families with experience of the program. The volunteers appreciated having received information in their training regarding medical equipment they might see and medical terms and acronyms they might hear. Regular supervision sessions were also noted as being valuable.

### 3.3. Challenges Experienced by Volunteer Biographers Engaged in the Story Project

The challenges identified by the volunteer biographers were grouped into seven themes, pertaining to (1) delays, (2) rapport/interaction, (3) family distress, (4) logistics, (5) transcribing-specific issues, (6) finding the themes/story, and (7) finalizing the biography. These themes, theme descriptions, and sample quotations are shown in Table 2.

### 3.4. Perceived Benefits of the Story Project Identified by Volunteers

The perceived benefits of the Story Project, as perceived by the volunteer biographers, were grouped into four main themes: (1) beneficial processes for families, (2) benefits for the volunteers, (3) intrinsic value of the final product, and (4) use of the product. These themes, theme descriptions, and sample quotations are shown in Table 3.

### 3.5. Most Recent Completed Projects

The six volunteers each reported on their most recent completed project. The time taken to complete work on one or more biography books with a family ranged from 3 months to 20 months, with most taking 12 months or less. In three cases, the volunteer biographer helped produce one biography book for the family—two of which were intended for the parents, and one was intended for the patient. Three of the volunteers reported producing two separate biography books for the family; in two cases, these included a book for parents and a book for siblings. In the third case, the volunteer biographer helped produce one book for the parents and a separate book for the patient. Five of the six volunteer biographers reported that the patient was alive when the biography was commenced. In three cases, the biographers reported that the patient was involved in the biography process. The nature of the patient’s involvement was different in each case, namely, contributing to interviews, providing written content, and audio-recordings (recorded by the mother) of the child talking about their life. Most of the biographies elicited information from immediate and extended family members, and in some cases, significant others (e.g., carer, friend, teacher).

Volunteer biographers mostly provided the same satisfaction rating out of 10 when rating their satisfaction with the process of working towards completing a biography, as compared with their satisfaction with the final product. All ratings were between 8 and 10, with three volunteers rating their satisfaction with both the process and outcome as 10/10.

## 4. Discussion

The current literature review, coupled with preliminary feasibility data, suggests that biography writing may be a valuable approach for use in the pediatric palliative care context. The current study provided new data for a biography writing project (the Story Project) for patients and families linked to a pediatric palliative care service. The flexibility of the Story Project enables families to decide how the biographical process and ultimate biography document could be most effectively utilized by the patient, parents, or siblings. To our knowledge, the Story Project is the first biographical approach to be used with well siblings as they navigate life with a seriously unwell brother or sister and subsequently in bereavement. The data obtained from the current study provide valuable information regarding the potential challenges and benefits described by volunteer biographers, which may help shape the development of similar biography programs at other sites.

### 4.1. Training

In line with the first aim of this study, data were elicited about the adequacy of the training used to prepare the volunteers for their role as biographers within a pediatric palliative care context. This is important given that it has been flagged that there is a dearth of information in the literature about the adequacy with which volunteers are trained for various roles in the palliative care context [40] despite the challenging and emotional context [41]. The current pilot study found satisfaction with training to be very high amongst the volunteer biographers, both in terms of the length and content of the training. The volunteers expressed that it would have been helpful to have had a clearer expectation of the lengthy time-frame that biography writing could take, and the training of future volunteer biographers has been revised accordingly. It was also suggested that future volunteer training could involve information shared by experienced volunteer biographers and families who have been involved with the Story Project.

Although the current study did not assess the competence with which the volunteers carried out their roles, the volunteers themselves did not convey a sense of unpreparedness for the palliative care context. Instead, they described feelings such as “To have been invited in to this family’s world is such a privilege” (ID6) and “The training well anticipated what was required for the role.” (ID3). It is documented in the literature that satisfaction with volunteer training enables volunteers to feel better able to handle situations related to terminal illness and death [42].

### 4.2. Challenges Experienced by the Volunteer Biographers

In line with the second aim of this study, the volunteers provided feedback about the challenges they had experienced in their role as biographers in the pediatric palliative care context. Six areas of challenge were identified, namely, (1) delays in the biography process, (2) establishing rapport and optimizing interactions with the patient/family, (3) working within the context of the family’s distress, (4) logistics, (5) transcribing-specific issues, (6) finding the themes/story, and (7) finalizing the biography.

The most frequent challenge reported by the volunteers pertained to various delays encountered in the biography process. While all the volunteers recognized that delays were understandable due to the competing demands experienced by families caring for a seriously ill child, they nevertheless reported sometimes experiencing a sense of disappointment when sessions were cancelled, losing a sense of continuity and flow in the project. In the event of prolonged delays, potentially due to fluctuations in the patient’s condition or even their death, volunteer engagement activities may be beneficial to support the volunteer and to facilitate volunteer retention in the program [43]. The excellent level of volunteer retention achieved in the Story Project may in part be attributable to supervision meetings with the volunteer manager and meetings every 2–3 months with the whole team of volunteer biographers and volunteer managers, with guest speakers and discussion about progress and challenges.

Challenges noted by the volunteers in the interview process included optimizing interactions with families and working within a context of high levels of family distress. This is significant, as it has been documented in the adult palliative care context that volunteers experience a particular challenge if not feeling confident when communicating with patients or feeling unable to ‘do enough’ for these patients [41]. Volunteers in the current study expressed a mindfulness for utilizing effective interview strategies, such as not interrupting the flow of the story by asking questions and balancing biography-related discussions with general chat. Although this may be challenging, they reported feeling adequately equipped to conduct these interviews.

In addition to various logistical challenges, such as technical issues and the sub-optimal space for interviewing, several other difficulties were identified. These included challenges in transcribing and finding themes within the stories. Often with many hours of interview content to transcribe and assimilate, these processes were found to be quite time-consuming. Factual stories shared may offer insights into underlying thoughts and emotions [19] and themes inferred. Stories can provide a “window” into the inner experiences of patients and their families [19]. However, the biographer needs to work with the patient or family member to clarify and ensure that they have a clear understanding of the most important themes.

The volunteers also described challenges related to finalizing the biography books. Although some of these challenges pertained to practical logistics of gathering photos and approvals, others related to self-doubt and anxiety about what the family will think of the final biography. Given that volunteers do not receive financial payment for their work, they commonly place high value in their work being appreciated [41]. This may contribute to a sense of nervousness when presenting the final products to families.

As the Story Project becomes more well-established and new volunteers enter the program, many of the challenges described in this study may be minimized through updates to the training and a sharing of experiences between experienced and new volunteers. This social dynamic between experienced and new volunteers has been identified in the literature as a key strategy for nurturing and retaining new volunteers at a time when they are least confident in their role [44].

### 4.3. Perceived Benefits Reported by the Volunteer Biographers

Consistent with the third aim of this study, four broad themes pertaining to the benefits of the Story Project were identified based on information provided by volunteer biographers, namely: (1) beneficial processes for families, (2) benefits for the volunteers, (3) the intrinsic value of the final product, and (4) utilization of the final biography book. Similar to what has been reported in the adult biography literature [3,6], the volunteers in the current study reported a range of benefits for patients or family members associated with the process of reflecting and sharing their story. Focusing on positive stories and what was of importance to them helped patients and families find meaning at a time when complex clinical care requirements were a big part of their lives. When caring for a seriously ill child, parents tend to put their own needs last [45]. Consequently, being asked to talk about their experiences and journey with their child was perceived to be a beneficial experience for parents. For example, one volunteer noted “I see the relief for families and friends telling their stories to a non-judgmental stranger” (ID3).

The volunteers themselves reported experiencing numerous benefits from their role in the Story Project. Although the palliative care context may be an emotionally difficult area to work in, the volunteer biographers in this study expressed that it was a privilege to engage with families and have them share their stories with them. This sense of privilege is a theme that has also emerged in other studies of volunteering in the palliative care context [6,46]. More specific personal benefits were also occasionally reported by the volunteers in the current study, such as becoming a better listener and enjoying the creativity of the project. In a national review of volunteering in Australia, it was noted that in the absence of receiving financial remuneration, volunteers value receiving social, human, and psychological capital in the form of personal enrichment, self-actualization, self-expression, and a sense of group accomplishment [47].

The volunteers identified an intrinsic value in achieving the final biography books. They expressed sharing a sense of pride with families in the co-creation of the biography books. As the books were typically created as a legacy document to reflect the life of the child with a life-limiting condition, the final product held special value for families: “a memento for them to enjoy forevermore” (ID2). Just as legacy objects such as an impression of an infant’s hand or foot may symbolically “eternalize” memories [48], so too may a written document or biography book capture experiences and stories as lasting legacies beyond the lifetime of the patient [49].

How patients or families chose to utilize the biography book in the pediatric palliative care context was regarded by the volunteer biographers as valuable. The biography books were considered helpful in facilitating sibling understanding and consolidating their memories of their brother or sister. Siblings also used the biography books as a way to talk and share with their peers about the life and death of their brother or sister. This is important in light of the many challenges experienced by well siblings, particularly in sharing their experiences of having a seriously ill brother or sister with peers [50]. One study noted that well siblings needed “an allowing space to talk about their experience” of having a seriously ill sibling [51]. Showing a biography book, written in age-appropriate language, provides a tool for facilitating such discussions.

The volunteer biographers also spoke of how the biography books could be well utilized by the patients’ parents. The volunteers noted that the biography document would be “a memento for them to enjoy forevermore” (ID2), enabling reflection of the positive experiences rather than just a recollection of the clinical care challenges. Further, as with siblings, the biography book may serve as a tool that parents use for communication and discussion with others, providing “A way of highlighting the person and his or her interests, relationships, moments and life force” (ID4). This is likely to be of importance given the commonly reported feelings of social isolation among parents of children with a life-limiting condition [52,53] or following the death of their child [54,55].

### 4.4. Data on Recent Projects

In line with the fourth aim of this study, this study elicited specific information and details regarding each volunteer’s most recent biography. The number of interview sessions carried out in the Story Project for each patient/family was greater than for all other adult [5,6] or child [28,35] biographical programs reviewed in this paper. Biographical interventions conducted with adult palliative care patients need to be brief due to the brevity of their remaining life, as adult patients are generally referred for palliative care much closer to the time of death than is the case for children who are referred for palliative care [56]. Moreover, in situations where parents are the primary contributor of information, the biography writing process is not time-limited and may continue beyond the death of the child.

Whereas most adult biographical programs in the palliative care context elicit information only from the patient [5], the volunteer biographers in the Story Project gave the patient or family the opportunity to involve various family members or significant others. The involvement of numerous informants adds complexity to the interview and write-up process, with one biographer noting “… if there are many interviews or lengthy interviews, extracting relevant content becomes challenging.” (ID3). However, the involvement of different people has the potential to add depth to the biography, drawing on different interactions and experiences shared with the patient. Given the time-consuming nature of a multi-informant approach, it may be challenging to achieve this using paid clinical health professionals. In contrast, trained volunteers may more easily fit into this holistic, family-centered approach to biography writing.

### 4.5. Limitations and Future Directions

The sample pool from which recruitment was possible was small (n = 6), albeit with 100% recruitment being achieved. Nevertheless, the respondents were highly engaged with the Story Project and provided extensive responses and rich descriptions about their experiences of the project. Given the small number of participants, we have provided a large number of quotations in the Results to give a clearer understanding of the nature and breadth of the themes identified.

It should be acknowledged that the current study described the experiences of a particular cohort of self-selected volunteers who have a willingness to work in the palliative care context. More specifically, the volunteers involved in the Story Project were retired individuals, mostly female and mostly from well-educated backgrounds, with these demographics being common among volunteers in the palliative care [57,58]. It is not known how volunteers from other demographics would find involvement in a program such as this one.

As more pediatric palliative care services implement the Story Project, future research with larger sample sizes is possible. It would be valuable to assess the experiences of patients and family members involved in the Story Project, in addition to the experiences of volunteers. Interviews with families could shed light on any challenges they encountered during their involvement with the project, what benefits they feel stemmed from the project, and how they subsequently used the completed biography books. As this is, to our knowledge, the first biography program that has the flexibility to target the needs of well siblings, it would be valuable to elicit sibling feedback about the interview process (if they were involved) and how they utilized the final biography book. As more biography documents are completed by patients, parents, and siblings as part of the Story Project, it would be of interest to analyze the key themes in these biographies, thereby highlighting ways in which individuals make meaning from their difficult circumstances. Finally, future research is needed to assess how these biography documents impact on the bereavement processes of parents and siblings.

## 5. Conclusions

Biography writing is an approach that holds much promise for the pediatric palliative care context to support patients and family members. A review of the literature highlighted the potential value that biography writing approaches may hold in the palliative care context for minimizing existential distress and enabling patients and/or family members to find meaning in difficult circumstances. The Story Project is described as a new biographical approach for use in the pediatric palliative care context. Pilot data from volunteer biographers reflected good program feasibility with a range of potential benefits.

## Figures and Tables

**Table 1:** Key steps in preparing biographies with families and patients known to the Pediatric Palliative Care Team through the Story Project.

Biography Phase	Key Steps
Volunteer training	Volunteer biographers receive 2 days training prior to being matched with a family. Training addresses the following key topics: pediatric palliative care, cultural diversity, spirituality, grief, self-care, biography writing, communication skills, ethical issues, and interview strategies. Code of conduct is explained and signed by volunteers, outlining issues such as confidentiality.
Matching and planning	Biographers are matched to families considering personality, commonalities, and geography (where both live). Biographers are introduced to the family by the volunteer manager. A preliminary discussion is held about the process of the biography and who the biography document will be for (e.g., parents, young siblings, patient) and who may be contributing to the interviews. The possibility of two books being completed can be part of this discussion (e.g., one for siblings, one for family). A consent form is signed by the volunteer biographer to agree to confidentiality and the disposal of all working materials at the completion of the biography. A consent form is also explained and completed by the patient and/or their parent/caregiver. Consent options are for copies of the biographies to be shared (i) for training of future volunteers, (ii) with other prospective families, and (iii) for ongoing education or quality improvement of volunteer biographers. The consenting process also highlights that volunteer biographers are mandatory reporters and therefore will need to report any content pertaining to this policy. The consent form outlines that the hospital network has the right to edit destructive or defamatory content and that the family provide a safe environment for the volunteer biographer.
Interview sessions	Biographers meet with the relevant family member(s) at a location convenient to the family (e.g., home, hospital, or respite/hospice service) or online. Interviews with the patient or family are generally not longer than 1 h in duration and are audio-recorded. Additional time is allowed for informal rapport building before and after interviews. Biographers use broad questions to invite family members to share their story. ‘Prompts’ can be used to commence the storytelling, but minimal interruptions are used that may interrupt the flow. At the start of each new session, the biographer recaps the content documented from the previous session, providing the patient or family member with an opportunity to correct as needed. With the family’s permission, biographers may interview other family members or family friends and include content from those interviews in the biography. The total number of interview sessions with the family/patient/friends will vary (typically ranging between 5–11 sessions), depending in part on whether one or two unique biography books are being created, the number of interviewees involved, and the way in which the family/patient shares their story.
Between sessions	Between each session, biographers re-listen to the audio-recordings and transcribe the interviews. Biographers are trained in using transcription software (*ExpressScribe*), but they may opt to transcribe manually from recordings. Biographers consider whether themes are emerging. Biographers consider whether they need further information in particular areas to help understand and be able to present the story. Biographers meet with the volunteer manager for supervision sessions to debrief, discuss progress, and discuss any challenges after each interview session. Story Project meetings with all biographers and volunteer managers are held every 2–3 months for ongoing educational purposes and to foster a sense of teamwork.
Writing and editing	An over-arching principal of biography writing is to use an authentic voice (including language idiosyncrasies), sharing the perspectives of the patient and family without altering or coloring their stories in the editing process. The biographer allows their story to be presented in whatever way the patient and family want to share it. It is the goal that to read it is to hear their voice. The biographer considers what the child would like to be remembered, and although a discussion of symptoms and treatments may be a necessary part of their story, the biographer will discuss with the family how much medical detail they want to include, encouraging families to maintain a focus on the personhood of the child.
Design	Discussions are held between the family and biographer at various times during the interview sessions about the order and layout of the biography book. Families are invited to provide photographs or artworks that might be used with the biography. A graphic designer is available once all the biography content has been completed to add elements of color and design, drawn from the content of the book and inspired by the personality of the child/young person.
Completion	A final draft is provided to the patient and/or the family who may wish to revise some details. When revisions are completed and printed, the final document is presented to the patient/family by the biographer and volunteer manager. The family usually receives 2 bound copies of the book and a USB containing an electronic version. The USB allows the families to add further life stories if they wish to do so in the future.

**Table 2:** Themes regarding the challenges experienced by volunteer biographers and sample quotations.

Themes (Theme Descriptions)	Sample Quotations
Delays (large gaps or appointment cancellations between interview sessions or delays in being able to achieve completion of the biography)	“Large gaps between interviews, dragging out the process” (ID2) “Delays (again very understandable). But they do “dislocate” the process”. (ID5) “Cancelled sessions… you feel a real let down… if there are many cancellations you sort of lose connection with the project” (ID6) “delays [in finalizing project] which means that momentum is lost.” (ID4) “… the reality of the chaos of life with a’ medical child’ needs to be recognised and understood.” (ID6)
Rapport/interaction (creating an environment in which the family members were comfortable and able to share their story)	“How to create an atmosphere of trust…” (ID3) “Remembering to just listen and not ask questions… a question can disrupt the flow of what the interviewee wants to tell you” (ID3) “Staying ‘on task’/’on track’, redirecting away from general conversation/chatting” (ID6)
Family distress (working in a context where families may be quite distressed)	“many competing demands on their time and energy” (ID4) “… [parent] was always (understandably) traumatised/shattered.” (ID5) “Assessing what is OK in terms of subject’s debriefing/outpouring, but remembering the role—our task—balancing the two. Sensing when time—a space—needs to be made for emotional process.” (ID6) “considering where the interviewee will be emotionally, am I prepared for all possibilities?” (ID3) “working with children in palliative care and their families- often an emotional space to be in.” (ID4)
Logistics (practical challenges encountered)	“Worrying that I will not be able to hear the recording… interview space is not always a quiet one!” (ID3) “Some locations (for example hospital setting) provide extra challenges… competing needs and priorities” (ID4) “technology was my biggest challenge” [in transcribing/editing phase] (ID1) “Editing could be improved with the use of Track Changes so amendments can be approved/ignored” (ID2)
Transcribing-specific challenges (challenges related to the transcribing process)	“Transcribing is time-consuming” (ID2) “Transcribing can be both monotonous -you have already heard what you are typing.” (ID3) “sometimes it is challenging to transcribe exactly what the interviewee said” (ID3) “Mumbling, fast or quiet speaking” (ID6)
Finding the themes/story (challenges related to formulating the themes and underlying story of the biography)	“Recognising what is relevant”. (ID6) “The challenge here is to not rush it—ponder it and let the story emerge” (ID3) “identifying themes for the biography” (ID3) “Once your theme emerges, how will you cover that theme, who do you need to interview and what do you need to ask?” (ID3) “… if there are many interviews or lengthy interviews, extracting relevant content becomes challenging.” (ID3)
Finalizing (challenges related to completing the biography document, including editing, design aspects, and delivering the final product)	“Family editing—Do they have time to read it?” (ID3) “Finalising the story. The necessary process of collaboration, approvals and sourcing photos…” (ID4) “Parents have told you about design aspects they would prefer but they are buried in interviews. How to find them is the challenge.” (ID3) “Presenting the biography to the family—The challenge? Will they like it? Will it meet their expectations? Self doubt!” (ID3)

**Table 3:** Themes regarding perceived benefits of the Story Project as identified by volunteer biographers.

Themes (Theme Description)	Sample Quotations
Beneficial process for families (including the process of reflecting, sharing, being listened to, taking time out from day-to-day challenges, and focusing on positive stories)	“Parents coming out of their shells, starting the process quite reserved/shy, but blossoming with more confidence” (ID2) “gives parents the opportunity to tell the story of their child to someone who is sincerely interested” (ID2) “children who are verbal can tell their own stories in their own way and they may feel more comfortable talking to a ‘stranger’ than a parent” (ID2) “I see the relief for families and friends telling their stories to a non-judgmental stranger.” (ID3) “The process itself often feels like positive timeout from the medical story and day to day difficulties.” (ID4) “… giving the families the opportunity to take time out to reminisce and go through old photographs seems like a gift…” (ID2) “The positive involvement of most/all contributors.” (ID5) “Family/individuals have felt heard, validated, appreciated.” (ID6) “The young person and family members all get an opportunity to focus on and hear positive stories about themselves- this is sometimes surprising and often moving.” (ID4)
Benefits for the volunteers (various personal gains, being entrusted with the family’s stories, a sense of doing something meaningful)	“I am a better listener than I have been in the past.” (ID3) “I enjoyed the creativity involved” (ID1) “The look on the recipient’s face (or recipients’ faces) made it entirely worthwhile.” (ID1) “providing them with the biographies to capture the essence of their lives in words and beautiful photographs is so rewarding.” (ID2) “This work feels both meaningful and a privilege.” (ID4) “To have been invited in to this family’s world is such a privilege, and to have helped them in some way is so rewarding.” (ID6) “I see honouring a short life by writing their story as a privilege.” (ID3) “… being part of a great team of biographers… and being led by a leader who is supporting, empathetic, cares that we are well trained for the task and has well considered solutions to difficult situations has been highly rewarding” (ID3) “A wider understanding is gained of the lives of children in palliative care and their families.” (ID6)
Value of the final product (achieving a legacy, something that families feel proud of)	“finished product… a source of pride for families” (ID2) “there is pride in creating a meaningful record and when people receive the hard copies of the book, in my experience this is a special joyful event and celebration.” (ID4) “I see the delight for families when they receive the biography—I see that they have had confirmed that their child’s life, be it short or restricted, had meaning.” (ID3) “Family has a book, or books that provide a legacy.” (ID6) “The opportunity to produce a quality product, to commemorate their precious child…” (ID1)
Use of product (including various beneficial ways in which patients, siblings, and parents have been able to use the completed biography)	“I wanted to give him [young sibling] the language to talk about death and ceremony with his peers. He felt comfortable to take his book to school. That was amazing” (ID1) “To be able to create a memory aid for siblings (some who may have never known their sibling) seems important.” (ID3) “to ‘show off’ to their friends and family—somebody has cared enough to go to’ all that trouble’ to put together a memento for them to enjoy forevermore” (ID2) “A way of highlighting the person and his or her interests, relationships, moments and life force.” (ID4)

## Data Availability

Raw data supporting the conclusions in this article will be made available by the corresponding author on reasonable request.
